# 

**Published:** 1884-04

**Authors:** 


THE SOUTHERN DENTAL ASSOCIATION.—The sixteenth annual session will be held in Lexington, Kentucky, on the first Tuesday in May, 1884. Ample arrangements have been made with all the railroads and hotels, for reduced rates.

The meeting promises to be one of unusual interest.

---

ILLINOIS STATE DENTAL SOCIETY.—The twentieth annual meeting of this society will be held at Springfield, Illinois, beginning Tuesday, May 13th, 1884, and continuing four days,

J. W. WASSALL, D. D. S., *Sec'y*.

### Index to Volume XV11.---Third Series.

A Remarkable Case - - - - - - - 47
A Simple Method of Producing Local anæsthesia - - 96
A Rare Case of Exostosis - - - - - - - 94
A Consideration of Some of the Causes which give Rise to Dental Decay - - - - - - - - 178
A Singular Case - - - - - - - - 333
A Rare Case of Exostosis - - - - - - - 188
A Variety of Leptothrix Discovered by Dr. W. D. Miller - - 269
A Few Practical Observations on the Treatment of the Pulp 272
Alimentation Among Savage and Civilized Peoples - - 289
Another Death from Chloroform - - - - 288
Another Opinion on Celluloid - - - - - 278
A New Work on Dental Medicine - - - - - 383
Aluminum Alloy - - - - - - - - 331
Amalgam Fillings - - - - - - - - 83
Anomalies in Eruption of the Teeth of Man - - - 372
Alumni Meeting - - - - - - - - 474
A Resume of the Different Preparations of Gold Used for Filling Teeth 448
Annual Commencement of the Dental Department of the University of Maryland - - - - - - - 520
Alveolar Abscess - - - - - - - - 49
An Appeal for a National Dental Association - - - 565
A New Steel - - - - - - - - 571
Best Method of Producing Anæsthesia - - - - - 527
Bone Degeneration in the Insane - - - - - 334
Bromide of Ethyl the most Perfect Anæsthetic for Short Operations 224
Be Tolerant - - - - - - - - - 186
Bone and Brain Disease in Syphilis - - - - - - 46
BIBLIOGRAPHICAL.
Dental Medicine - - - - - - - 476
United States Pharmacopœtia - - - - - - 332
Conservative Treatment of the Pulp - - - - - - 433
Case of Antral Abscess of Twenty Years Standing - - 375
Caries Dentium - - - - - - - - 525
Correlated Diseases ot the Teeth and Ear - - - - 1
Change of Time of Meeting of Southern Dental Association - 48
Continuous Gum Work - - - - - - - 56
Calcareous Deposits - - - - - - - 182
Chromic Acid as a Caustic - - - - - - - 48
Dentistry and Otopathy - - - - - - 486

Dental Legislation - - - - - - - 425
Dies, Etc. - - - - - - - - 423
Danger—Carelessness - - - - - - - 478
Death in the Dental Chair - - - - - - 471
Dental Nomenclature - - - - - - 337
Dr. Genese's Articulator - - - - - - 344
Dental Association Meetings - - - - - 236
Dental Caries - - - - - - - - 77
Dental Formation in the Nasal Cavity - - - - 96
Dental Caries - - - - - - - - - 126
Death from Dichloride of Ethidene - - - - 48
Dr. Genese's Method of Treating Fractured Jaw - - - 44
Dental Legislation—Opinions for and against - - - 568
Dental Students in Baltimore City - - - - - 380
Dental Department of the University of California - - 379
Ear-ache in Children - - - - - - - 189
Education of Mothers - - - - - - 319
Efficacy of Vaccinations - - - - - - 139
Exposed Pulp and Treatment - - - - - 175
Effect of Tobacco on Youth - - - - - 287
Effects of Malarial Poisoning on the Dental Pulp - - 465
Etiology of Dental Caries - - - - - - 497
Food Makes the Man - - - - - - - 331
Form and Nature of Accidents Occasioned by the Eruption of Wisdom Teeth - - - - - - - 365
Foreign Bodies in the Air Passages - - - - 94
General Anæsthesia Scarcely Justifiable in Extraction of Teeth - 207
Gluten - - - - - - - - - 144
Hardening Plaster Casts - - - - - - - 95
Hardening Steel - - - - - - - - 190
Hardening Dental Rubber in Steatite Without the Aid of Flasks 469
How to Keep Cool - - - - - - - 144
How do Parasites Produce Disease - - - - 335
Hot Water as a Restorative in Chloroform Narcosis - - 239
Inebriety of the Teeth - - - - - - - 141
Infidelity Among Physicians - - - - - 138
Inodorous Iodoform - - - - - - - 240
In Difference of Dr. S. C. Barnum - - - - - 420
Illinois State Dental Association - - - - 571
Journalistic Change - - - - - - - - 139
Keeping the Teeth Clean - - - - - - 38
Longevity of Occupations - - - - - - 47
Maryland State Dental Association - - - - - 381
Maryland and Distric of Columbia Deneal Association - - 380
Mankind's Mistakes - - - - - - - 187
Mechanical Treatment of Cleft Palate, Obturators, and Artificial Velum 357

Metallic Crowns - - - - - - - 86
Meeting of Georgia State Dental Society - - - - 48
Metaillc Crowns - - - - - - - 13
Meeting of State Board of Dental Examiners - - - 20
Maryiand and District Columbia Association Meeting - - 235
New Discoveries - - - - - - - 240
Nocotine and its Action upon the Teeth - - - - 122
OBITUARY.
Dr. Robert Early - - - - - - - 47
Dr. Arthur C. Ford - - - - - - - 475
Dr. Warren Welch - - - - - - - 140
Prof. Thomas L. Buckingham, M. D., D. D. S. - - - 284
Dr. Henry Hobart Keech. M. D., D. D. S. - - - - 572
Our Dental Brethren - - - - - - - 95
Odonto-Chirurgical Society of London - - - - - 36
On Density of Enamel as an Element in Determing the Proper Time to Devitalize the Tooth Pulp - - - - - 170
Odontological Society of Pennsylvania - - - - - 456
On Certain Conditions of Dead Teeth - - - - - 280
Ozone as an Anæsthetic and Hypnotic - - - - - 328
On the Conservative Treatment of the Dental Pulp - - - - 230
Overcoming Heredity - - - - - - - 368
On the Relation between Dental Lesions and Disease of the Eye - 529
Practical Knowledge - - - - - - - 377
Practical Suggestions During Dentition - - - - - 190
Prof. Chisolm's Rules in Administering Chloroform - - - 41
Pericementitis—Its Manifestations and Effects Upon the General System 363
Pennsylvania State Dental Society - - - - - - 89
Pittsburgh Dental Association - - - - - - 136
Prone Position During Operations Upon the Jaws - - - 241
Proceedings of the American Dental Association for 1883 - - 244
Present Systems and the Impending Education - - - - 256
Personal Identity Established by the Teeth ; the Dentist a Scientific Expert - - - - - - - - 385
Periodontitis—Cause and Treatment - - - - 71
Pre-Natal Influence as Affecting the Teeth - - - - 559
Remedy For Inflameed and Sore Mouth, Caused by Wearing Artificial Teeth on Rubber Plates - - - - - 180
Recent Notices of Dr. Gorgas' Treatise on "Dental Medicine" - 481
Syphilis and Rachitis - - - - - - - 240
Should Anæsthesia be Resorted to for the Extraction of Teeth - 64
Steamer for Dental Purposes - - - - - - 348
Some Points in Oral Surgery of Interest to the General Practitioner 349
Southern Dental Association - - - - - - 88
Smoothness of Dental Plates - - - - - - 332
Successful Exsection of Inferior Dental Nerve - - - - 238
Simplified Treatment of Disease of the Dental Pulp - - 228

Staining Artificial Teeth to Match Exceptional Natural Teeth 517
Southern Dental Associations, Annual Meeting for 1883 - 134
Some Interesting Points in Oral Surgery - - - - 321
The Necessity of Uniform Legislation - - - - - 326
The Treatment of a Cold - - - - - - - 282
The Dental Institutions of Learning - - - - - 283
The University of Maryland - - - - - 237
The Odontological Society of Great Britain - - - 31
The Maryland State Dental Association - - - - - 330
The Care of the Teeth - - - - - - - - 91
The Composition and Effects of Chloral Hydrate - - - 89
The Application of Disenfectants and Escharotics in the Treatment of
Tartar and its Removal from the Teeth - - - 213
Tin Foil as a Filling - - - - - - - - - - 460
Treatment of Fracture of the Jaw, with Critical Remarks, as sent to
Prof. D. Hayes Agnew, M. D. - - - - - 443
Treatment of Children's Teeth - - - - - 506
The Causes of the Failure of Gold as a filling Material - - 514
The Maryland Dental Bill - - - - - - - 523
The Association Meetings for 1883 - - - - - 137
Things to be Considered in Regulating Teeth - - - 184
The Proposed Bill to Regulate the Practice of Dentistry in the State
of Maryland - - - - - - - 381
To Disguise the Odor of Iodiform - - - - - - 48
To Stop Hiccough - - - - - - - 143
To Disguise the Odor of Iodoform - - - - - - 143
The Effect of Nitrate of Silver on the Skin and Mucous Membrane 189
Transactions of the State Board of Dental Examiners - - 193
The Maryland Dental Bill - - - - - - - - 473
University of Maryland Dental Department - - - 474
Under the Surgeon's Touch - - - - - - - 93
The Southern Dental Association - - - - - 571
Transplantation of Muscle in Man - - - - - - 336
Treatment of Pulpless Teeth - - - - - - 276
Tempering Steel - - - - - - - - 187
The Use and Abuse of Amalgam - - - - - 113
The Teeth from a Medico-Legal Aspect - - - - 233
The Dental Bill of the State of New Jersey - - - 567
Visiting List for 1884—
Physician's Visiting List - - - 382
Physician's Daily Pocket Record - - 383
Medical Record, Visiting List, and Physician's Diary 383
Views about Decay of the Teeth—Old and New - - - 26
What is Vitality of a Tooth - - - - - - 130
Zinc Dies for Swaging Plates - - - - - - 162

### To Readers and Correspondents.

Communications intended for publication, and exchange jou[illegible] papers should be addressed to the Editor of the American Jour[illegible] Dental Science, 259 N. Eutaw St., Baltimore, Md. All letter[illegible] to the business of the Journal, or containing cash remitt[illegible] directed to Messrs. Snowden & Cowman. Articles for publ[illegible] received by the editor at least three weeks previous to [illegible] on which the number in which it is designed for them to [illegible]

CONTENTS:

Art. IV.—On the Relations between Dental Lesions and Diseases o[illegible] By Henry Power, F. R. C. S.......................

Atr. II.—Pre-Natal Influences as Affecting the Teeth. By Geo. H. Cu[illegible]

Art. III.—An Appeal for a National Dental Association..............

The Dental Bill of the State of New Jersey...........

EDITORIAL, ETC.

Dental Legislation—Opinions For and Against.......................

A New Steel.................................................

The Southern Dental Association..........................................

Illinois State Dental Asssociation..........................................

OBITUBRY.

In Memoriam.—Henry Hobart Keech, M. D., D. D. S. By. R. G.........

In Memoriam.—Henry Hobart Keech, M. D., D. D. S. By F. J. S. G.......

☞ The American Journal of Dental Science will cont[illegible] pages of reading matter in each number, making a volume of about

*Six Hundred* pages,

EXCLUSIVE OF ADVERTISEMENTS.

THE

### AMERICAN JOURNAL

OF

### DENTAL SCIENCE.

The JOURNAL is issued on the first of each month and contains not less than *fifty* pages of reading matter in each number, exclusive of advertisements, making a volume of *six hundred* pages per annum.

In each number will be found Original Communications, Correspondence, Selected Articles, Monthly Summary, Bibliographical Notices and Editorial Matter, and every effort will be exerted to render the JOURNAL worthy of the patronage of the Dental profession.

A generous co-operation is respectfully solicited from the members of the profession; and as the JOURNAL has now a printing office of its own, which materially lessens the cost of its publication, it will be issued at the reduced subscription price of

**$2.50 Per Annum in Advance.**

Communications are respectfully solicited on all subjects pertaining to practice of dentistry, as well as reports of cases occurring in dental practice.

### RATES OF ADVERTISING.

| | 1 MO. | 2 MO. | 3 MO. | 6 MO. | 12 MO. |
|---|---|---|---|---|---|
| 1 PAGE. | $15 00 | $22 50 | $30 00 | $50 00 | $100 00 |
| ½ " | 10 00 | 15 00 | 20 00 | 30 00 | 50 00 |
| ¼ " | 7 00 | 11 00 | 15 00 | 20 00 | 30 00 |
| ⅛ " | 5 00 | 8 00 | 11 00 | 15 00 | 20 00 |

All original communications designed for publication, works for review, new instruments for notice, exchanges, &c., should be directed to the editor, 259 N. Eutaw St., Baltimore, Md.

All advertisements and subscriptions should be addressed to

SNOWDEN & COWMAN,

*Publishers of the Am., Journal of Dental Science,*

86 W. FAYETTE ST., BET., CHARLES & LIBERTY STS..

BALTIMORE, MD.

